# Aspergilli Response to Benzalkonium Chloride and Novel-Synthesized Fullerenol/Benzalkonium Chloride Nanocomposite

**DOI:** 10.1155/2015/109262

**Published:** 2015-07-29

**Authors:** Nikola Unković, Milica Ljaljević Grbić, Miloš Stupar, Jelena Vukojević, Vesna Janković, Danica Jović, Aleksandar Djordjević

**Affiliations:** ^1^Department of Algology, Mycology and Lichenology, Faculty of Biology, University of Belgrade, Institute of Botany and Botanical Garden “Jevremovac”, Takovska 43, 11 000 Belgrade, Serbia; ^2^Institute of Meat Hygiene and Technology, Kaćanskog 13, 11 000 Belgrade, Serbia; ^3^Department of Chemistry, Biochemistry and Environmental Protection, Faculty of Science, University of Novi Sad, Trg Dositeja Obradovića 3, 21000 Novi Sad, Serbia

## Abstract

A comprehensive comparative analysis of antifungal potential of benzalkonium chloride and newly synthesized fullerenol/benzalkonium chloride nanocomposite was conducted to assess the possible impact of carbon-based nanocarrier on antimicrobial properties of the commonly used biocide. Physical characterization of synthesized nanocomposite showed zeta potential of +37.4 mV and inhomogeneous particles size distribution, with nanocomposite particles' dimensions within 30–143 nm and maximum number of particles at 44 nm. The effect of pure and fullerenol nanocarrier-bound biocide was evaluated in eight *Aspergillus* species. In mycelial growth assay, nanocomposite was more potent, as fungicidal effect of 1.04/0.6 *μ*g mL^−1^ was obtained in all but one of the isolates (*A. niger*), while proportional concentration of pure biocide (0.6 *μ*g mL^−1^) completely inhibited mycelial growth of only three *Aspergillus* species. However, conidia appear to be less susceptible to nanocomposite treatment, as lower fungistatic (MIC) and fungicidal (MFC) concentrations were obtained with biocide alone (MIC in range from 0.03 to 0.15 *μ*g mL^−1^ and MFC from 0.075 to 0.45 *μ*g mL^−1^). To a different degree, both substances stimulated aflatoxin B1 production and inhibited ochratoxin A synthesis. Very low mycelium biomass yield, in range from 1.0 to 3.0 mg dry weight, was documented in both biocide and nanocomposite enriched medium.

## 1. Introduction

The health implications associated with indoor surface fungal proliferation have always been an important issue. However, only recently has the topic of the environmental fungal contamination and consequential health risk gained increased public concern. Generally, though not a norm, fungi are of interest only in water damaged buildings with excess moisture, possibly a result of leaks in the envelope, plumbing failures, or condensation. Under such conditions, exposure to indoor fungal growth can cause or exacerbate a variety of health problems, from minor allergic reactions and respiratory obstructions to severe systemic mycoses and mycotoxicoses. With more than 180 currently recognized species, fungi of the genus* Aspergillus* are one of the primary contaminants of indoor air and surfaces worldwide, as well as causative agents of a wide assortment of diseases collectively dubbed aspergilloses [1]. Given all the foregoing, removal and subsequent prevention of growth of these and other toxigenic and pathogenic fungi, nowadays, represent a significant challenge, as chemical treatments must be long-term and effective, without adverse effects on the consumers and the environment.

Since first introduced as antimicrobial agents by Domagk over seventy years ago, to date, many alterations to basic quaternary ammonium compounds (QACs) formulas have been made hoping to augment their already potent antimicrobial activity [2]. As a result, their application as surface cleaning agents in domestic household environments, foodservice industry, and pharmaceutical and healthcare institutions, to name a few, has increased considerably in recent years. Today, amongst the more prominent and favored QACs hygienic adjuncts in disinfectant cleansing formulations is benzalkonium chloride (BAC), an assorted mixture of alkyldimethylbenzylammonium chlorides of various even-numbered alkyl chain lengths [3]. In short, it is a cationic surface-acting quaternary ammonium compound, with mechanism of antimicrobial activity essentially based on disruption of cell membrane phospholipid bilayer and possibly disruption and denaturation of structural proteins and enzymes [4–6]. However, despite well-known and documented broad-spectrum antimicrobial activity (e.g., [7, 8]), development of microorganisms with decreased susceptibilities to BAC treatments (e.g., [9, 10]) is considered to be rising trend, prompting the need for some changes.

Fullerenols (C_60_(OH)_*n*_), polyhydroxyl water-soluble fullerenes, in form of polyanionic nanoparticles, have been examined in numerous* in vitro* and* in vivo* model systems [11, 12]. Special attention has been given to fullerenols with a large number of hydroxyl groups (*n* > 16), since they are nontoxic for the most of examined biological model systems, possess significant antioxidative properties [13], and produce radical oxygen species, such as singlet oxygen and superoxide, after UV photosensitization [14]. Experiments conducted on various aquatic organisms and yeasts have proven that fullerenol nanoparticles (FNP) stimulate growth and exhibit protective effects from heavy metal induced toxicity [15, 16].

In order to enable rapid, potent, and broad-spectrum antifungal activity, with the least possible negative impact on the consumer and the environment, as well as to avoid or minimize the risk of the development of the resistant fungal strains, it is of the utmost importance to look for new and alter extant fungicide formulations. In addition to understanding chemistry and distinct modes of action, proper and efficient application of fungicidal agents can only be achieved by extensive investigation of their biological and physiological properties. Therefore, a principal purpose of this research was to evaluate and compare* in vitro* antifungal effect of widely utilized nitrogen-based quaternary ammonium compound, benzalkonium chloride, and fullerenol nanoparticles-bound biocide BAC on morphophysiological traits of somatic and reproductive phase of eight toxigenic* Aspergillus* species isolated from the biodeteriorated wall paintings.

## 2. Material and Methods

### 2.1. Tested Fungal Isolates

Morphophysiological changes, induced by BAC, FNP, and fullerenol/benzalkonium chloride nanocomposite (FNP/BAC), were investigated on eight* Aspergillus* species isolated from the biodeteriorated mural paintings of the old Church of the Holy Ascension in Veliki Krčimir, Serbia (43° 5′ 28.2′′ N, 22° 12′ 14.4′′ E):* Aspergillus flavipes* (Bainier & R. Sartory) Thom & Church,* Aspergillus flavus* Link,* Aspergillus fumigatus* Fresen.,* Aspergillus nidulans* (Eidam) G. Winter,* Aspergillus niger* Tiegh.,* Aspergillus ochraceus* Wilh.,* Aspergillus parasiticus* Speare, and* Aspergillus terreus* Thom. Isolated fungi were deposited to the Mycotheca of the Department for Algology, Mycology and Lichenology, Institute of Botany and Botanical Garden “Jevremovac”, Faculty of Biology, University of Belgrade. Selected isolates were stored in cryovials with 1.5 mL of 30% glycerol at −75°C.

### 2.2. Conidial Suspensions

Conidial suspensions of selected fungi were prepared by washing conidia from the surface of the 7-day-old Malt Extract Agar (MEA) slants with sterile saline (NaCl 0.9%, HemofarmhospitaLogica) containing 0.1% Tween 20 (Sinex Laboratory). Using a hemocytometer (Reichert, Warner-Lambert Technologies) conidia were counted on a 1 mm^2^ surface and the concentrations of conidia in the suspensions were calculated per formula:

(1) $$\begin{array}{c} \text{Number of conidia}/\text{m}{\text{m}}^{2}\times 10\;000\times \text{dilution}. \end{array}$$

The conidial suspensions were adjusted with saline solution to the concentration of approximately 1.0 × 10^5^. The inocula were stored at −20°C and prior to experiments, dilutions of the inocula were cultured on solid MEA to verify the absence of contamination and check the validity of the inocula.

For mycotoxicological analysis, conidial suspensions were prepared per given procedure, with the exception of using sterile distilled water, instead of saline solution, in the preparation process.

### 2.3. Benzalkonium Chloride (BAC)

An aqueous solution of the quaternary ammonium compound, benzalkonium chloride (50 v/v), was obtained from a team of expert chemists and restorers of the Institute for Protection of Cultural Monuments in Serbia. Prior to experiments, biocide was diluted in sterile deionized water to make a stock solution of final concentration 3 v/v %.

### 2.4. Fullerenol (FNP) and Fullerenol/Benzalkonium Chloride Nanocomposite (FNP/BAC) Synthesis

Fullerenol C_60_(OH)_24_ was obtained by complete substitution of bromine atoms of polybromine derivative C_60_Br_24_ with hydroxyl groups [17, 18]. Thus, obtained powder was dissolved in water to prepare FNP solution of concentration of 0.125 mg mL^−1^, which was sonicated 15 min prior to further FNP/BAC nanocomposite preparation. To synthesize FNP/BAC nanocomposite, 6.93 mL of 5 v/v % BAC was mixed with 4.95 mL of FNP solution. Final concentrations of components in nanocomposite were as follows: 3 v/v % for BAC and 0.052 mg mL^−1^ for FNP. Nanocomposite mixture was stored in dark and was characterized as stable for more than several weeks. Size distribution of FNP and nanocomposite particles was measured after 48 h by Dynamic Light Scattering (DLS) on Zetasizer Nano ZS (Malvern UK).

For scanning electron microscopy, stable nanocomposite in water was evaporated under reduced pressure on Al substrate and was steamed with gold. Electron images were obtained using JEOL JSM 6460 LV microscope. Morphology and structure of FNP/BAC nanocomposite in solution were evaluated via atomic force microscopy (AFM). Surface topography and phase images were simultaneously acquired by standard AFM tapping mode using a commercial SNC (Solid Nitride Cone) AFM probe (NanoScience-Team Nanotec GmbH), with the tip radius lower than 10 nm. Highly orientated pyrolytic graphite (HOPG) was used as a surface. Multimode quadrex SPM with a Nanoscope IIIa controller (Veeco Instruments, Inc.), operated under ambient conditions, was used.

### 2.5. Mycelial Growth Assay

To elucidate the susceptibility of the somatic phase (mycelium) of tested isolates to BAC, FNP, and FNP/BAC nanocomposites, the modified macrodilution method, utilizing MEA nutrient media, was used [19]. The stock solutions of selected chemicals were further diluted in 5 mL of melted MEA in Petri plates (*Ø* 50 mm) to form final concentrations in range from 0.03 to 0.6 *μ*g mL^−1^, 0.05 to 1.04 *μ*g mL^−1^, and 0.05/0.03 to 1.04/0.6 *μ*g mL^−1^ for BAC, FNP, and FNP/BAC, respectively. The fungi were inoculated at the center of the MEA, via sterile cork borer. The inoculated plates were incubated in a thermostat at 25 ± 2°C for 21 days, during which the growth of fungal colonies was observed daily. In addition, on weekly basis, mycelial growth dynamics were monitored by means of colony diameter measurement. Mycelial growth inhibition (%) was calculated per formula of Pandey et al. [20]:

(2) $$\begin{array}{c} \text{Mycelial growth inhibition }\left(\;\%\;\right)=\frac{100\left(\text{dc}-\text{dt}\right)}{\text{dc}}, \end{array}$$

where dc is average diameter of fungal colony in control, while dt is average diameter of fungal colony in treatment.

### 2.6. Conidia Germination Assay

Sensitivity of the asexual reproductive stage of the fungal life cycle (conidia), to the BAC, FNP, and FNP/BAC treatment, was evaluated via modified microdilution technique [21, 22]. In the wells of 96-well microtiter plates (F-bottom, Ratiolab), different volumes of investigated substances were dissolved in Malt Extract Broth (MEB), in addition to 10 *μ*L of conidial suspension, to make the final concentrations in range from 20.1 to 0.03 *μ*g mL^−1^, 34.84 to 0.05 *μ*g mL^−1^, and 34.84/20.1 to 0.05/0.03 *μ*g mL^−1^ for BAC, FNP, and FNP/BAC, respectively. The microplates were incubated for 72 h at 25 ± 2°C. After incubation period, the lowest concentrations without visible growth, under a binocular microscope (Stemi DV4, Zeiss), were defined as the minimum inhibitory concentrations (MICs), concentrations that completely inhibited fungal growth. The minimum fungicidal concentrations (MFCs) were determined by serial subcultivation of 2 *μ*L into microtiter plates containing 100 *μ*L of MEB. The lowest concentrations with no visible growth were defined as the MFCs, indicating 99.5% killing of the original inoculums.

### 2.7. Mycotoxicological Survey

Among tested micromycetes, three well-known mycotoxin producers,* Aspergillus flavus*,* A. ochraceus,* and* A. parasiticus*, were chosen to study the effects of BAC, FNP, and FNP/BAC treatment on aflatoxin B1 (AFB1) and ochratoxin A (OTA) production. Selected isolates were cultivated in 250 mL of semisynthetic glucose medium (GPK) for 5 days at room temperature (22 ± 2°C) on a rotary shaker (Titramax 1000, Heidolph) set to 200 rpm. Prior to incubation, inoculated media were enriched with different volumes of tested substances to make final concentrations of 0.003 *μ*g mL^−1^ for BAC, 0.0052 *μ*g mL^−1^ for FNP, and 0.0052/0.003 *μ*g mL^−1^ for FNP/BAC. After cultivation period, mycelial biomasses were filtered through Whatman No. 4 filter paper, dried for 24 h at 70°C, and measured. Filtrates (100 mL) were dissolved in 150 mL of methanol (99.8%, Sigma-Aldrich), concentrated under reduced pressure in a rotary evaporator (Rotavapor R-210, Buchi) at 90°C to dryness, measured, and redissolved in fivefold greater mass of 70% methanol. Quantitative (ELISA) analyses of AFB1 and OTA in samples were performed per protocol of enzyme-linked immunosorbent assay kits Celer AFLA B1 (code MA220/MA221) and I'screen OCHRA (code OR360/OR361) (Tecna s.r.l.). The detection limit for both methods is 1 *μ*g kg^−1^ of given mycotoxins.

### 2.8. Microscopic Analysis

For microscopic observations of induced morphophysiological changes in tested microfungi, samples of mycelium and/or conidiogenous apparatus were stained with few drops of Crystal Violet or Lactophenol Cotton Blue, put on slides, and analyzed with optical microscope Zeiss AxioImager M1, using software AxioVision Release 4.6. Results were documented with original micrographs.

### 2.9. Statistical Analysis

The results were expressed as the mean ± standard error of data obtained from three measurements. To test any significant differences, one-way analysis of variance (ANOVA) was performed using Microsoft Excel 2010 (Office Professional Plus 2010). *p* values less than 0.05 were considered statistically significant.

## 3. Results

### 3.1. Fullerenol and Fullerenol/Benzalkonium Chloride Nanocomposite Characterization

**Figure 1:**
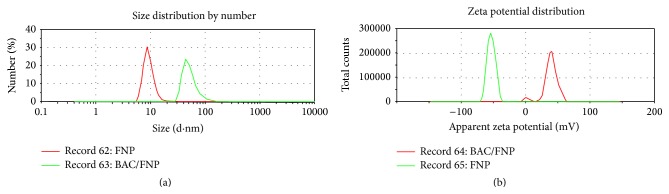
Size distributions by number (a) and zeta potentials (b) of fullerenol (FNP) and fullerenol/benzalkonium chloride (FNP/BAC) nanocomposite.

**Figure 2:**
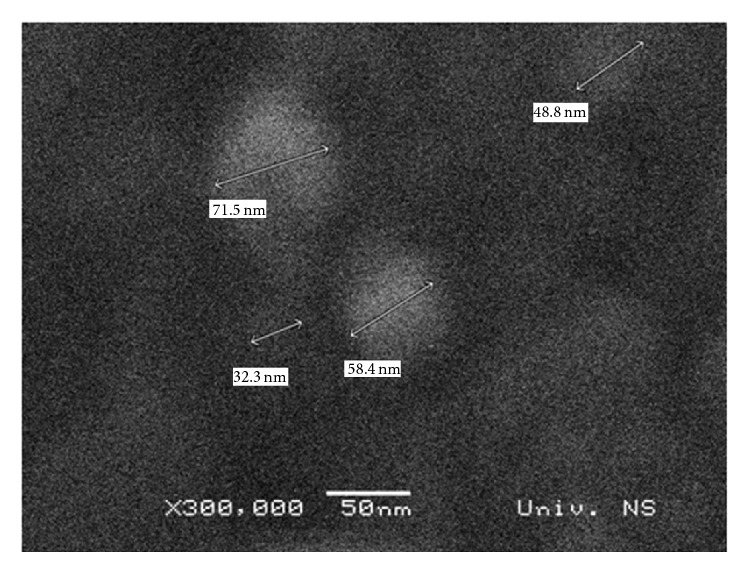
SEM image of fullerenol/benzalkonium chloride nanocomposite.

**Figure 3:**
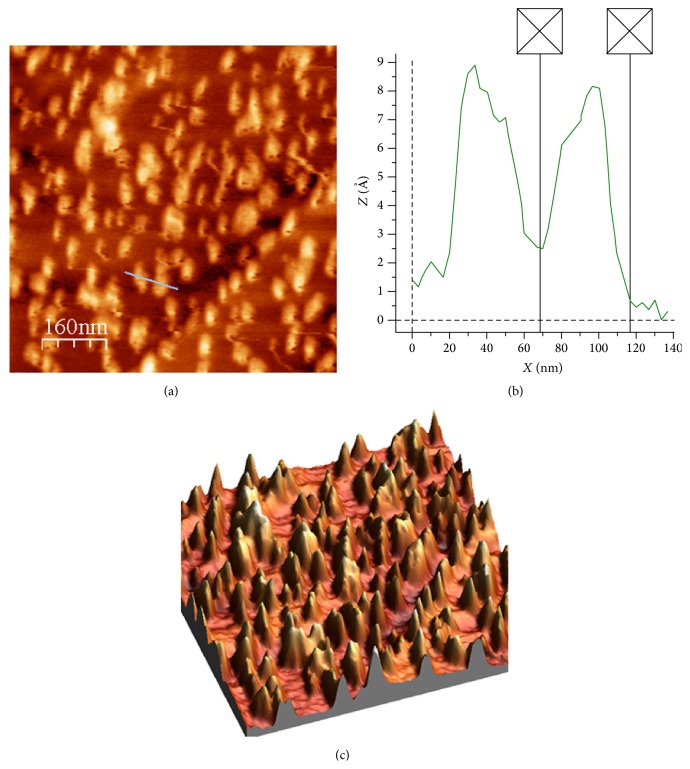
Water solution of fullerenol/benzalkonium chloride nanocomposite: large-scale image, 810 × 810 nm^2^ (a); corresponding cross section with two nanoparticles, 50 nm and 48 nm, and maximal peaks for the nanocomposite, 9 nm and 8 nm (b); 3D image of nanocomposite on the HOPG surface (c).

Size distributions by number and zeta potentials of FNP and FNP/BAC nanocomposite are presented in Figure 1. Size distribution results of examined samples indicate that samples are quite inhomogeneous, that is, polydisperse. FNP's size was within 6–16 nm, with the maximum number of particles (30%) at 8.7 nm. FNP/BAC size distribution shows that nanocomposite particles' dimensions were within 30–143 nm, with the maximum number of particles at 44 nm (23%). On SEM image (Figure 2), nanocomposite particles of 32.3, 48.8, 58.4, and 71.5 nm are shown, which correspond with the DLS measurements of FNP/BAC particle size distribution (Figure 1(a)). As can be seen from Figure 3, sample is quite inhomogeneous, composed mainly of nanoparticles between 30 and 90 nm, with larger particles formed by agglomeration of smaller ones. These results are in agreement with the conducted DLS and SEM measurements. Time-dependant agglomeration of nanoparticles was detected neither in FNP solution nor in nanocomposite. Zeta potential of FNP was −53.3 mV, while zeta potential of FNP/BAC nanocomposite was +37.4 mV. These results are consistent with previously published findings on the distribution by number and electric charge of fullerenol nanoparticles [23, 24]. Formulated nanocomposite was stable at 40°C in the dark for 30 days.

### 3.2. Benzalkonium Chloride and Fullerenol/Benzalkonium Chloride Nanocomposite Induced Mycelial Growth Inhibition

**Figure 4:**
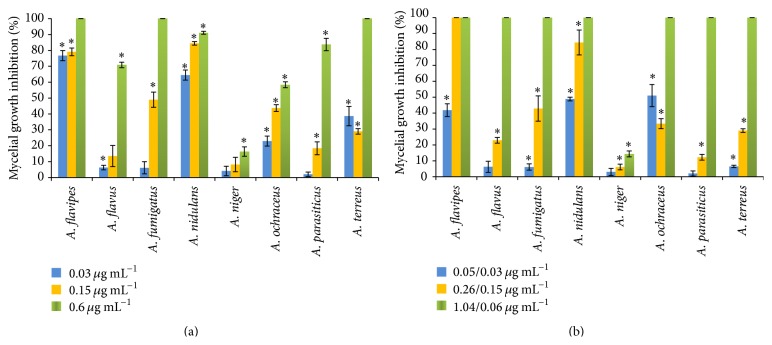
Mycelial growth inhibition in 21-day-old* Aspergillus* spp. cultures treated with benzalkonium chloride concentrations of 0.03 *μ*g mL^−1^, 0.15 *μ*g mL^−1^, and 0.6 *μ*g mL^−1^ (a) and fullerenol/benzalkonium chloride concentrations of 0.05/0.03 *μ*g mL^−1^, 0.26/0.15 *μ*g mL^−1^, and 1.04/0.6 *μ*g mL^−1^ (b). Results are expressed as means ± standard error of values obtained from triplicate experiments (^*∗*^ *p* < 0.05).

Different susceptibility levels of tested isolates to biocide BAC and FNP/BAC nanocomposite treatments were documented in mycelial growth assay (Figure 4). In both cases, the most resistant isolate was* Aspergillus niger*, with inhibition of mycelium growth observed in range from 4.08 ± 2.97% to 16.33 ± 2.97% in BAC treatment and between 3.06 ± 2.22% and 14.29 ± 1.86% in FNP/BAC treatment. Compared to the pure biocide, fullerenol bound form was more potent, as fungicidal effect of 1.04/0.6 *μ*g mL^−1^ was obtained in all of the tested isolates, with the exception of* A. niger*. On the other hand, proportional concentration of pure biocide completely inhibited mycelial growth in only three fungal species (*A. flavipes*,* A. fumigatus,* and* A. terreus*). Furthermore,* A. flavipes* was also the most sensitive isolate, with mycelium growth completely suppressed at 0.26/0.15 *μ*g mL^−1^ of FNP/BAC.

With increase in both BAC and FNP/BAC concentrations, a higher percentage of mycelial growth inhibition was achieved (Figure 4). In most cases, regardless of the concentration used, gradual reduction of inhibitory effect during cultivation period was observed. Consistent inhibition of mycelial growth, throughout 21-day incubation period, was only recorded in* A. flavus* (6.25 ± 1.39% and 6.25 ± 0.79% at 0.03 *μ*g mL^−1^ of BAC and 0.05/0.03 *μ*g mL^−1^ of FNP/BAC, resp.) and* A. parasiticus* (2.04 ± 1.43% at 0.03 *μ*g mL^−1^ of BAC). On the other hand, no inhibition of mycelium growth and/or morphophysiological changes were observed in any of the FNP treated cultures.

**Figure 5:**
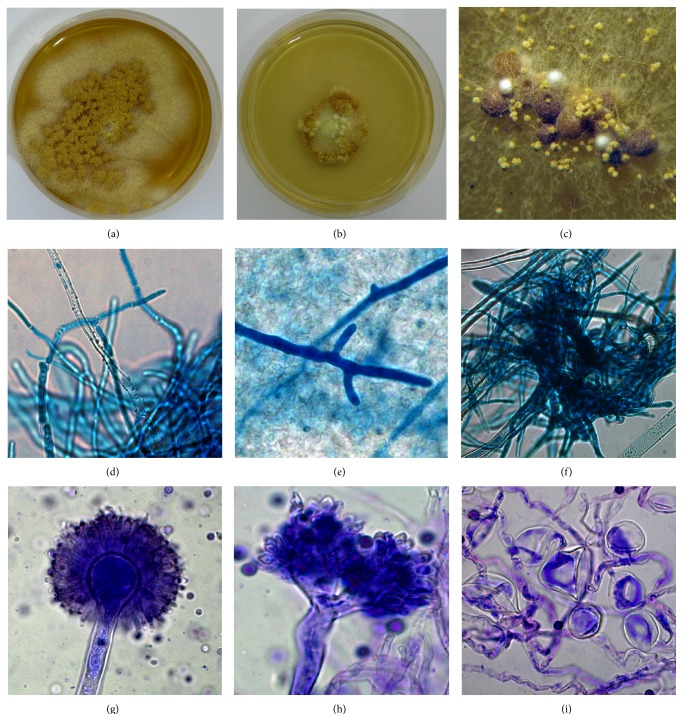
Morphophysiological alterations in 21-day-old* Aspergillus ochraceus* culture (MEA) induced by benzalkonium chloride (0.6 *μ*g mL^−1^): control colony (a); altered colony morphotype (b);* in situ* sclerotia (×20) (c); typical hyphal morphology (control, ×630) (d); conspicuous “worm-like” hyphae in mycelial mass (×400) (e); initial stage of sclerotium formation in form of clamps of fragmented, thick-walled hyphae (×630) (f); normal conidial head (control, ×630) (g); bifurcate conidiophore with aberrant conidial heads (×630) (h); and distorted hyphae with intercalary and apical swellings (×630) (i).

In addition to inhibited growth,* A. ochraceus* colonies, grown in the presence of 0.15 *μ*g mL^−1^ of benzalkonium chloride, were consistently characterized by apparent morphological variations, compared to control (Figure 5). Such changes included altered colony morphology, with dominance of sterile area and restricted sporulation, development of aberrant conidiophores and conidial heads, presence of “worm-like” hyphae, and anomalies in form of extensive swellings along the distorted hyphae. Compared to control, treated colonies were distinguished by central sterile area containing tufts of aerial mycelium and circumferential sporulation (Figure 5(b)). The alterations in hyphal structures included presence of voluminous, thick-walled, branched, coenocytic “worm-like” hyphae often embedded in a mycelium mass of distorted hyphae with irregular intercalary and apical swellings (Figures 5(e) and 5(i)). Both changes correlated with sterile area of altered colony. Abnormal conidiogenous apparatus, with distorted “two-headed” conidiophores, collapsed, irregularly shaped ellipsoidal vesicles bearing poorly developed phialides with conic apices and enlarged conidia, were also common (Figure 5(h)). Initial phase of sclerotium formation, that is, clamps of fragmented, thick-walled hyphae, as well as fully formed ocher, spherical sclerotia of different size, partially covered with aerial mycelium, were likewise observed (Figures 5(f) and 5(c)). None of the above-described morphological alterations were documented for any of the other treated* Aspergillus* species.

### 3.3. Conidia Susceptibility to Benzalkonium Chloride and Fullerenol/Benzalkonium Chloride Nanocomposite

High fungistatic and fungicidal activities were obtained in conidia germination assay for both BAC and FNP/BAC nanocomposite, as demonstrated by very low MIC (in range from 0.03 to 0.15 *μ*g mL^−1^ for BAC and 0.08/0.045 to 0.52/0.3 *μ*g mL^−1^ for FNP/BAC) and MFC (in range from 0.075 to 0.45 *μ*g mL^−1^ for BAC and 0.13/0.075 to 0.52/0.3 *μ*g mL^−1^ for FNP/BAC) values (Table 1). Lower MIC values were documented for pure biocide in all but one of the investigated isolates (*Aspergillus flavipes*), whereas MFC values, although generally lower for BAC, were the same or higher as in FNP/BAC treatment for three of the eight tested microfungi (*A. flavipes*,* A. flavus,* and* A. parasiticus*). Isolates* A. nidulans*,* A. ochraceus,* and* A. terreus* were the most susceptible to BAC treatment, with fungicidal effect achieved at 0.075 *μ*g mL^−1^, while the most resistant isolate was* A. parasiticus* (MFC 0.45 *μ*g mL^−1^). In regard to FNP/BAC,* A. flavipes* was the most sensitive isolate and growth was completely suppressed with 0.13/0.075 *μ*g mL^−1^. On the other hand, five tested microfungi displayed the same, highest level of resistance to FNP/BAC treatment (MFC 0.52/0.3 *μ*g mL^−1^) (Table 1). Distinct morphological variations, in form of visible loss of conidia melanization (albino phenotype) and a significantly lower number of conidial heads, were observed in* A. niger* and* A. flavipes* cultures grown in the presence of FNP/BAC concentrations close to MIC (0.13/0.075 and 0.1/0.06 *μ*g mL^−1^, resp.). Reinoculation of albino colonies, via single spore transfer, onto the sterile MEA resulted in formation of colonies with typical species morphology, suggesting reversibility of induced morphophysiological changes. No statistically significant inhibition of conidial germination (*p* < 0.05) was observed for any of the isolates cultured on MEB enriched with pure FNP nanoparticles; hence, no data regarding FNP are presented in Table 1.

### 3.4. Mycotoxin and Biomass Production in Benzalkonium Chloride, Fullerenol, and Fullerenol/Benzalkonium Chloride Nanocomposite Treated Cultures

**Figure 6:**
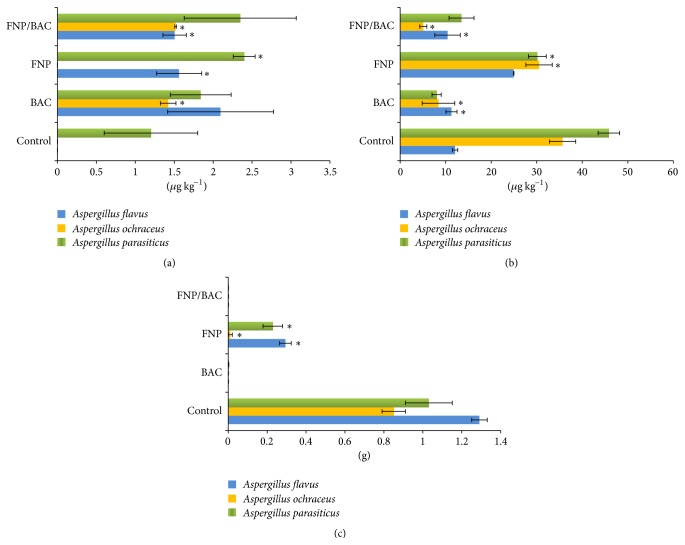
Aflatoxin B1 (a), ochratoxin A (b), and biomass (c) production in toxigenic* Aspergillus flavus*,* Aspergillus ochraceus,* and* Aspergillus parasiticus* cultures treated with biocide benzalkonium chloride (BAC), fullerenol nanoparticles (FNP), and fullerenol/benzalkonium chloride nanocomposite (FNP/BAC). Results are expressed as means ± standard error of values obtained from triplicate experiments (^*∗*^ *p* < 0.05).

The results of biomass production and quantitative AFB1 and OTA analyses are presented in Figure 6.* Aspergillus flavus* and* A. ochraceus* control samples were characterized by the lack of and/or immeasurable quantities (<1 *μ*g kg^−1^) of AFB1, while in* A. parasiticus* detectable mycotoxin level was present (1.201 ± 0.6). In general, investigated substances, to a varying degree, stimulated AFB1 synthesis in all but one case. Apparently, FNP has no effect on aflatoxin B1 production in* A. ochraceus*, conclusion further supported by approximately the same concentrations of AFB1 detected in both BAC (1.422 ± 0.1) and FNP/BAC (1.518 ± 0.01) treated* A. ochraceus* cultures. On the other hand, in* A. parasiticus*, FNP and FNP/BAC induced nearly twofold higher AFB1 production (2.399 ± 0.14 and 2.347 ± 0.72, resp.) compared to the control and roughly 30% higher than pure biocide. However, in* A. flavus* BAC stimulated AFB1 production the most (2.095 ± 0.68), whereas the concentrations in FNP (1.562 ± 0.29) and FNP/BAC (1.505 ± 0.15) were smaller and practically indistinguishable.

Unlike AFB1, ochratoxin A, in range from 12.08 ± 0.58 to 45.85 ± 2.35, was detected in all control samples. Likewise, opposite to the aflatoxin B1, used treatments in* A. parasiticus* and* A. ochraceus* cultures resulted in considerable reduction of OTA concentrations. In* A. ochraceus*, FNP reduced OTA concentration slightly (30.51 ± 2.9), while BAC and FNP/BAC treatments caused significant inhibition of ochratoxin A synthesis (8.43 ± 3.58 and 5.09 ± 0.77, resp.). Similar results, with FNP being the weakest (30.15 ± 1.95) and BAC (8.01 ± 1.01) and FNP/BAC (13.48 ± 2.73) the strongest OTA inhibitors, were documented in* A. parasiticus*. On the other hand, in BAC and FNP/BAC treated* A. flavus* cultures, OTA concentrations were in the level of the control (12.08 ± 0.58). Interestingly, however, FNP treatment did not inhibit but stimulated OTA production twofold (24.95 ± 0.05), a trait, based on the results, more closely associated with AFB1 than OTA metabolism.

Mycelium biomass yield ranged from 0.851 ± 0.06 to 1.291 ± 0.04 g dry weight in the controls and 0.011 ± 0.01 to 0.294 ± 0.03 g in the FNP treatment, while very low production, in range from 0.001 ± 0.0 to 0.003 ± 0.0 g dry weight, was documented in both BAC and FNP/BAC enriched medium (Figure 6(c)).

## 4. Discussion

Strong antifungal activity of biocide BAC and FNP/BAC nanocomposite, estimated via microdilution and macrodilution assays, suggests pronounced capability of tested agents to interfere with various stages of the fungal life cycle. Obtained results demonstrate that even low concentrations of pure and nanocarrier-bound benzalkonium chloride can hinder conidia germination and achieve a high percentage of mycelium growth inhibition. In mycelial growth assay, proportional concentrations of BAC (0.6 *μ*g mL^−1^) and FNP/BAC (0.6 *μ*g mL^−1^ of BAC bound to 1.04 *μ*g mL^−1^ of FNP) had different levels of impact, since nanocomposite induced fungicidal effect in all but one of the tested isolates (*Aspergillus niger*), while BAC merely inhibited growth of the majority of the isolates. The mechanisms thought to be responsible for nanocomposite's increased toxicity against fungi may be linked with C_60_(OH)_*x*_-triggered process of programmed cell death, caspase-dependent apoptosis, characterized by chromatin condensation, activation of caspases, and fragmentation of DNA without membrane breakdown,* inter alia* [25]. Furthermore, fullerenols can easily enter into the excited state, in the presence of visible or UV light, and in a series of successive reactions produce ^1^O_2_ and O_2_^•^ [26]. Subsequently, ^1^O_2_ can start a chain reaction in which a more dangerous reactive oxygen species is produced, resulting in cell damage, disruption in the DNA chains, and cell death [27]. When exposed to subfungicidal concentrations of BAC and FNP/BAC, complete fungistatic effect was only achieved in two closely related species from the* Aspergillus* section* Flavi* (*A. flavus* and* A. parasiticus*), while in other tested isolates, fungal growth evidently continued midst the incubation period, albeit at a much slower rate. Fungi are apparently able to cope with BAC and FNP/BAC's very low dose induced toxicity and gradually, over time, entirely suppress biocides inhibitory effect on mycelium growth. In both treatments, the most resistant isolate was* A. niger*, on whom fungicidal effect was not achieved at all. However, this is not unexpected as fungal melanins, abundant in cell walls of this fungus, have a protective role and are probably responsible for the higher resistance documented. As for* A. ochraceus*, complete mycelium growth inhibition (100%) was not accomplished even with the highest concentration of BAC used in the experiment (0.6 *μ*g mL^−1^), although observed morphophysiological variations indicate interference of biocide with fungal cellular metabolism. Aberrant changes in somatic (extensive swellings and squashed, distorted hyphae) and reproductive structures morphology (distorted conidiophores with collapsed, irregularly shaped vesicles bearing poorly developed phialides) were most likely a result of partial dissociation of cellular membrane phospholipid bilayers, caused by the biocides disruption of intermolecular interactions or denaturation/inactivation of structural proteins and enzymes essential for the developmental processes [4–6]. On the other hand, development of thick-walled “worm-like” hyphae, fully matured sclerotia, and formation of additional conidial heads per conidiophore in some instances, is a defense mechanism that ensures fungal survival in the adverse environmental conditions, incurred due to biocide presence.

As demonstrated by the very low MIC and MFC values, both BAC and FNP/BAC possess strong potential to interfere with the first step in the fungal asexual life cycle, that is, conidia germination. However, higher fungistatic and fungicidal activities, for example, up to tenfold for* A. terreus*, were obtained for BAC alone, pointing out potential protective role of fullerenol nanoparticles in conidia germination process and/or possible decline in the BAC biocidal activity due to its linkage to nanocarrier. On the other hand, in three of the eight tested isolates, nanocomposite fungicidal activity was equal (*A. flavus*) or higher (*A. flavipes* and* A. parasiticus*), compared to pure biocide, emphasizing the already well-known significant role of individual fungal physiology in species-specific susceptibility to biocide treatment. Inhibition of melanin synthesis and subsequent formation of* A. niger* and* A. flavipes* albino phenotype in cultures treated with subinhibitory FNP/BAC concentrations are a nanocomposite property most likely associated with the nanocarrier, rather than biocide, given the fact that fullerenol nanoparticles were already known to induce depigmentation of the melanized conidiogenous apparatus in* A. niger* [28]. Melanins mediate fungal survival during adverse environmental conditions [29] and are, likewise, recognized as virulence factor for pathogenic fungi, known to affect the immune response of the host [30]. Albeit not essential for fungal growth, when present, strong melanization results in pathogens increased resistance to host antimicrobial mechanisms, as well as biocides and other antimicrobial treatments. As such, even though nanocomposite showed weaker antifungal effect in majority of the isolates, compared to pure biocide, FNP/BAC induced inhibition in melanin production, proven to greatly reduce the pathogenic power of melanized fungi [31], substantially boosts its potential for application as antimicrobial agent in disinfectant cleansing formulations.

Up to date, numerous studies have demonstrated the role of various stress factors in mycotoxin production of many filamentous fungi (e.g., [32]). In this regard, accurate assessment оf investigated biocides fungistatic and fungicidal activity is a major concern, given that wrong estimation, and ensuing usage of subinhibitory biocide doses can result in not only mould survival but also enhanced mycotoxin production [33]. However, it is important to note that stimulation of mycotoxins production and growth are not always mutually exclusive [34]. In study presented here, as expected, biomass yield of selected* Aspergillus* species in glucose-rich liquid medium was severely inhibited by suboptimal inhibitory biocide and nanocomposite concentrations, yet only AFB1 concentrations were notably increased. Apparently, used BAC and FNP/BAC concentrations, although much lower (25–50x and 50–100x for biocide and nanocomposite, resp.) compared to documented MIC values, nonetheless, created stress conditions responsible for the increased AFB1 production, probably a form of a defense mechanism. Furthermore, such results are fully consistent with earlier findings that low fungicide doses stimulate mycotoxin deoxynivalenol (DON, vomitoxin) production in* Fusarium* species [33]. On the other hand, in both treatments, OTA concentrations surprisingly reduced in comparison to the control. Considering that OTA concentrations did not increase, as was expected due to harmful environmental conditions, such discrepancies in the results can only be attributed to the isolates genetic predispositions [35]. At any rate, both BAC and FNP/BAC seem to strongly influence mycotoxin production. Still, due to interspecific variations in AFB1 and OTA concentrations, produced in the investigated treatments, no unambiguous conclusion as to which one is more potent can be made. It appears that complex interactions occurring between biocides and isolates, as well as ecological and developmental factors, that exert influence at the transcriptional level and enhance/quench the expression of the biosynthetic genes play crucial role in this process. Concerning health issues, low concentration-induced AFB1 production is rather problematic, since aflatoxins are considered the most carcinogenic, mutagenic, and teratogenic substances of biological origin [36]. It was recently demonstrated that AFB1 has lower skin permeability rate than OTA, but dermal exposure, via contaminated surfaces, to the DNA-reactive and genotoxic AFB1 can result in serious health risks and should be avoided [37].

## 5. Conclusions

Several studies to date have been engaged in research on anti-*Aspergillus* properties of nitrogen-based quaternary ammonium compound, that is, benzalkonium chloride (e.g., [38, 39]). However, to our knowledge, this is the first report on utilizing commonly used biocide and polyhydroxylated water-soluble fullerene derivative, fullerenol C_60_(OH)_24_, in form of polyanionic nanoparticles, to synthesize stable nanocomposite and evaluate its antifungal potential on selected* Aspergillus* species. Our research confirms well-established antimicrobial activity of BAC but also emphasizes greater potency of FNP/BAC nanocomposite in suppression of fungal somatic growth. Moreover, even though nanocomposite treatment had a weaker effect on conidia germination, reduction of fungal pathogenicity, via inhibition of melanin synthesis, is a feature that should not be overlooked. We also point out the need for careful evaluation of BAC and FNP/BAC doses, before application, as treatments with subinhibitory concentrations of both substances stimulate aflatoxin B1 production. In conclusion, the preliminary results of our study suggest that novel-synthesized nanocomposite might be used as an effective antifungal alternative to benzalkonium chloride in treating various fungal contaminated surfaces. Further in-depth investigations on common contaminants from other fungal genera, as well as prospective evaluation of safety, will be necessary prior to* in situ* application.

## Figures and Tables

**Table 1:** Minimum inhibitory concentration (MIC) and minimum fungicidal concentration (MFC) for benzalkonium chloride (BAC) and fullerenol/benzalkonium chloride nanocomposite (FNP/BAC) obtained in microdilution method.

Tested isolates	BAC		FNP/BAC	
	MIC (*μ*g mL^−1^)	MFC (*μ*g mL^−1^)	MIC (*μ*g mL^−1^)	MFC (*μ*g mL^−1^)
*Aspergillus flavipes *	0.045	0.15	0.08/0.045	0.13/0.075
*Aspergillus flavus *	0.075	0.15	0.26/0.15	0.26/0.15
*Aspergillus fumigatus *	0.15	0.15	0.52/0.3	0.52/0.3
*Aspergillus nidulans *	0.075	0.075	0.26/0.15	0.26/0.15
*Aspergillus niger *	0.15	0.15	0.52/0.3	0.52/0.3
*Aspergillus ochraceus *	0.075	0.075	0.52/0.3	0.52/0.3
*Aspergillus parasiticus *	0.15	0.45	0.52/0.3	0.52/0.3
*Aspergillus terreus *	0.03	0.075	0.52/0.3	0.52/0.3
